# Pancreatic neuroendocrine tumors in children and adolescents—Data from the German MET studies (1997–2023)

**DOI:** 10.1111/jne.70039

**Published:** 2025-05-11

**Authors:** Katharina Karges, Marina Kunstreich, Ulrich‐Frank Pape, Jörg Fuchs, Christian Vokuhl, Michael Abele, Dominik T. Schneider, Ines B. Brecht, Constantin Lapa, Michael C. Frühwald, Peter Vorwerk, Antje Redlich, Michaela Kuhlen

**Affiliations:** ^1^ Pediatrics and Adolescent Medicine, Faculty of Medicine University of Augsburg Augsburg Germany; ^2^ Department of Pediatrics, Pediatric Hematology/Oncology Otto‐von‐Guericke‐University Magdeburg Germany; ^3^ Department of Internal Medicine and Gastroenterology Asklepios Klinik St. Georg, Asklepios Tumorzentrum Hamburg Germany; ^4^ Department of Pediatric Surgery and Pediatric Urology University Children's Hospital, Eberhard‐Karls University Tuebingen Germany; ^5^ Section of Pediatric Pathology, Department of Pathology University Hospital Bonn Bonn Germany; ^6^ Pediatric Hematology and Oncology University Children's Hospital, Eberhard‐Karls University Tübingen Germany; ^7^ Clinic of Pediatrics Dortmund Municipal Hospital, University Witten/Herdecke Witten Germany; ^8^ Nuclear Medicine, Faculty of Medicine University of Augsburg Augsburg Germany; ^9^ Bavarian Cancer Research Center (BZKF) Augsburg Germany

**Keywords:** children and adolescents, neuroendocrine tumors, outcome, pancreatic, treatment

## Abstract

Pancreatic neuroendocrine tumors (panNETs) are rare pediatric malignancies with age‐specific clinical and biological features. Data on their presentation, management, and outcomes remain limited. This retrospective study analyzed 28 pediatric panNET cases from the German Malignant Endocrine Tumor (MET) Registry enrolled between 1997 and 2024. Clinical presentation, diagnostics, and treatment were evaluated to identify prognostic factors and outcomes. The cohort included 18 females (64.3%) and 10 males (35.7%), with a median age at diagnosis of 14.7 years. Nonfunctional tumors predominated (75%). Genetic syndromes were identified in 17.9% of patients. Localized disease showed a 3‐year overall survival (OS) of 100%, while metastatic disease had a 3‐year OS of 50.9%. Event‐free survival was significantly associated with the presence of distant metastases (M0 vs. M1, *p* = .0082) and complete surgical resection (R_0_ vs. R_1/2_ vs. no resection, *p* = .0077) but not with lymph node involvement (N0 vs. N1, *p* = .12), tumor localization within the pancreas (head vs. body vs. tail, *p* = .86), the extent of the primary tumor (pT1‐2 vs. pT3‐4, *p* = 1.0), pathological grade (G1 vs. G2‐3, *p* = .28), or proliferation index (Ki67 ≤ 10% vs. >10%, *p* = .11). This study underscores the importance of disease stage and surgical resection as key prognostic factors in pediatric panNETs. It highlights the need for pediatric‐specific management guidelines, integration of genetic screening, and expanded molecular profiling to optimize outcomes for children and adolescents with panNETs.

## 1 INTRODUCTION

Pancreatic neuroendocrine tumors (panNETs) are a rare subset of neoplasms arising from the neuroendocrine cells that derive from gastrointestinal stem cells that originate from the endoderm.^1^, ^2^ While panNETs represent only 1%–2% of all pancreatic tumors, their occurrence in children and adolescents is even more infrequent, with markedly distinct clinical, biological, and prognostic characteristics compared to adult presentations.^3^, ^4^

In children and adolescents, panNETs constitute a clinically significant yet uncommon malignancy.^5^, ^6^ The annual incidence is extremely low, estimated at less than 0.1 per million individuals.^7^ These tumors exhibit heterogeneous behavior, classified as functional (hormone‐producing) or nonfunctional, with the latter often detected incidentally or presenting with nonspecific symptoms.^8^

Among pediatric panNETs, insulinomas predominate, representing 50%–60% of functional tumors and are characterized by hypoglycemic symptoms due to excessive insulin production. Gastrinomas follow, constituting 20%–30% of functional panNETs and are associated with Zollinger–Ellison syndrome. Less common functional variants, including glucagonomas, VIPomas, and somatostatinomas, each contribute less than 10% to the overall functional tumor landscape.^9^, ^10^

The etiology of pediatric panNETs remains largely elusive.^8^ However, associations have been documented with specific hereditary syndromes, including multiple endocrine neoplasia type 1 (MEN1), von Hippel–Lindau (VHL) disease, neurofibromatosis type 1 (NF1), and tuberous sclerosis complex (TSC).^11^, ^12^, ^13^ Among these, MEN1 is the most commonly associated syndrome, accounting for approximately 10%–25% of pediatric panNETs.^5^, ^12^

Survival outcomes for pediatric panNETs demonstrate significant variability depending on tumor characteristics, stage, and therapeutic interventions. The surveillance, epidemiology, and end Results (SEER) database indicates that localized pediatric panNETs exhibit a promising 5‐year overall survival (OS) rate exceeding 80%. Conversely, metastatic cases present a more challenging prognosis, with survival rates ranging from 30% to 50%, depending on disease progression and treatment response.^3^, ^14^

Contemporary management strategies encompass a comprehensive approach, including surgical resection for localized tumors and advanced systemic therapies such as chemotherapy, somatostatin analogs, targeted interventions, and peptide receptor radionuclide therapy (PRRT) for metastatic cases.^15^

The complexity of pediatric panNETs necessitates a nuanced, multidisciplinary management strategy with sustained long‐term monitoring to address potential recurrence and posttreatment endocrine complications.^16^

Given the limited epidemiological data and unique clinical challenges, this study aims to comprehensively analyze a cohort of pediatric and adolescent panNET patients from the German Malignant Endocrine Tumors (MET) Registry. By examining clinical characteristics, diagnostic methodologies, treatment, and outcomes, we seek to elucidate key prognostic factors and therapeutic strategies. Ultimately, our research aspires to develop evidence‐based guidelines for optimizing pediatric panNET management.

## 2 PATIENTS AND METHODS

This retrospective cohort study analyzed data from children and adolescents under 18 years of age with histopathologically confirmed panNETs, enrolled in the German MET Registry between January 1, 1997, and June 30, 2024. The final follow‐up was completed by August 31, 2024. All diagnoses were confirmed through centralized reference evaluation.

Data were collected from participating centers in Germany, Switzerland, and Austria, including demographics, clinical presentation, diagnostic approach, treatment modalities, and outcomes. Frequencies are reported relative to the number of patients with available data.

Inclusion criteria were: (1) histopathologically confirmed panNET (World Health Organization (WHO) classification at time of diagnosis), (2) age <18 years, (3) NET grades 1–3 of pancreatic origin, and (4) available medical records. Patients with other endocrine tumors were excluded. The study adhered to the Declaration of Helsinki and was approved by the Institutional Review Boards (IRBs) of the University of Luebeck (IRB 97125), Otto‐von‐Guericke‐University Magdeburg (IRB 174/12 and 52/22), Germany, and all participating institutions. Informed consent was obtained from patients or legal guardians.

Diagnosis included clinical, biochemical, imaging, and histopathological assessments. Functioning tumors were defined by the presence of a clinical syndrome and corroborating hormonal abnormalities; immunohistochemistry alone was not considered sufficient for diagnosis. Further diagnostic details are available in Table S1. Information on hereditary cancer predisposition syndromes was collected when available. However, systematic genetic testing was not mandatory and may not have been conducted or reported for all patients.

### 2.1 Treatment of pancreatic neuroendocrine tumors

Surgical resection was the mainstay treatment, tailored to tumor size, location, stage, and spread. Chemotherapy and other systemic treatments were employed in selected cases of progressive unresectable or metastatic disease. The GPOH‐MET 1997 protocol included general treatment recommendations, while more recent updates (GPOH‐MET 2013 and MET Registry) emphasized individualized treatment planning.

### 2.2 Statistical analysis

Descriptive statistics were used to summarize the data. Kaplan–Meier analysis estimated overall (OS) and event‐free survival (EFS), with subgroup comparisons by log‐rank test. Statistical analyses were performed using R software, with significance set at *p* < .05.

## 3 RESULTS

### 3.1 Demographics and clinical characteristics of 28 children and adolescents with pancreatic NET

We identified 28 patients with panNETs diagnosed during childhood or adolescence. The study cohort demonstrated a female predominance, with 18 females (64.3%) and 10 males (35.7%). The median age at diagnosis was 14.7 years (range: 6.2–17.9 years). Among the cohort, five patients presented with documented tumor predisposition syndromes. In the remaining 23 patients, no such syndromes were reported; however, for 18 of these, no information on genetic testing was available.

The median time between initial symptom manifestation and definitive diagnosis showed considerable variation, with a median duration of 1.3 months (range: 0–25 months). Notably, one patient (3.8%) from the 26 informative cases was diagnosed incidentally.

The clinical presentation was characterized by diverse symptomatology. Abdominal and/or back pain emerged as the predominant initial symptom, affecting 21 of 26 patients (80.8%); for the remaining 2 of 28 patients, data were not available. Additional presenting features included weight loss in 9/26 patients (34.6%; missing information in two patients), palpable abdominal mass in 6/23 (26.1%; missing information in five patients), and metabolic imbalances in 5/25 (20.0%; missing information in three patients).

Functional panNETs were identified in 5 of 23 patients (21.7%; missing information in five patients). Within this subgroup, insulinomas constituted the majority with four cases, while gastrinoma was diagnosed in one patient. Comprehensive demographic data and clinical characteristics of the study cohort are presented in Table 1.

**TABLE 1:** Demographics and clinical characteristics of 28 patients with pancreatic neuroendocrine tumors.

Characteristics	German MET registry	
	Count	Proportion, %
Total number of patients (*n*)	28	100
Age at diagnosis, years		
<10	2	7.1
10–14	15	53.6
15–18	11	39.3
Median age at diagnosis, years	14.7 (range, 6.2–17.9)	
Sex		
Male	10	35.7
Female	18	64.3
Cancer predisposition		
MEN1, pathogenic variant	3	10.7
MEN1, no pathogenic variant	5	17.9
Tuberous sclerosis	1	3.6
von Hippel–Lindau syndrome	1	3.6
Unknown	18	64.3
Performance status at diagnosis		
Not impaired/mildly impaired	15	53.6
(Severely) Impaired	8	28.6
Unknown	5	17.9
Presenting symptoms		
Abdominal/back pain	21/26	
Weight loss	9/26	
Palpable abdominal resistance	6/23	
Metabolic imbalance	5/25	
Icterus	4/25	
Vomiting	4/26	
Diarrhea	4/26	
Hepatomegaly	4/24	
Cholestasis	2/8	
(Sub‐)ileus	1/25	
Skin discoloration	1/25	
No symptoms/incidental finding	1/26	
Unknown	2/28	
Functional/nonfunctional panNET		
Insulinoma	4	14.3
Gastrinoma	1	3.6
Nonfunctional	18	64.3
Unknown/unclear	5	17.9
Preoperative diagnostics		
Chest CT scans	9	32.1
Bone scintigraphy	5	17.9
Somatostatin receptor‐based imaging	12	42.9
[^111^In]In octreotide scintigraphy	5	17.9
[^68^Ga]Ga‐DOTATATE PET/CT	11	39.3
[^18^F]‐FDG PET/CT	5	17.9

### 3.2 Initial diagnostics in 28 children and adolescents with pancreatic NET

**FIGURE 1:**
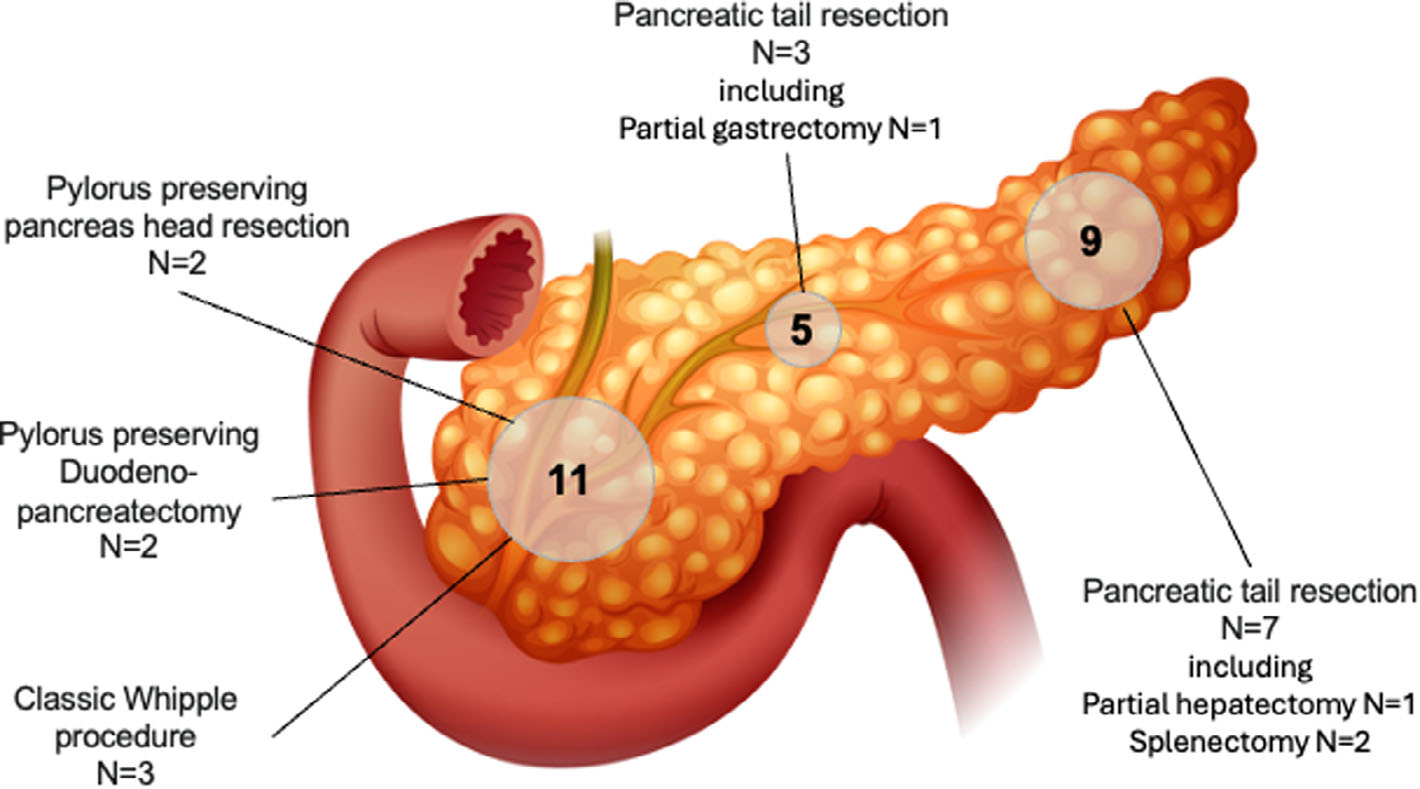
Anatomical distribution of pancreatic neuroendocrine tumors and corresponding surgical interventions in pediatric and adolescent patients. The schematic illustration depicts tumor localization within different anatomical regions of the pancreas (head, body, and tail) and the surgical procedures performed based on the anatomical site. Tumor localization data were not available for three patients. Surgical resection was performed in 18 patients from the total cohort, with details available in 17.

The diagnostic evaluation utilized multiple imaging modalities for tumor identification and characterization. Abdominal sonography was the most frequently employed imaging technique (*n* = 21), closely followed by magnetic resonance imaging (MRI) (*n* = 20). Computed tomography (CT) was utilized in seven patients, while endosonography was performed in two patients, yielding diagnostic findings in one case. Postsurgical imaging with ^68^Ga DOTA PET/CT was conducted in one patient. The median tumor diameter was 4.0 cm (based on imaging), with sizes ranging from 1.6 to 16.0 cm. The anatomical distribution of tumors within the pancreas is depicted in Figure 1.

Initial disease staging revealed localized disease in 10 of 26 patients (38.5%; missing information in two patients) and isolated lymph node involvement (cN1) in 2 of 26 patients (7.7%; missing information in two patients). Distant metastases (cM1) were present in 15 of 27 patients (55.6%; missing information in one patient) at diagnosis, including one patient with supraclavicular lymph node involvement only. The liver represented the predominant site of metastatic disease (*n* = 13), while additional metastatic involvement was documented in the skeletal system (*n* = 2) and ovary (*n* = 1), and isolated in the lungs (*n* = 2).

Among the patients with lymph node metastases (*n* = 2), one had a grade 1 tumor, and the other had a grade 2 tumor. For the patients with distant metastases (*n* = 15), tumor grade was available in 14 cases: six had grade 2, eight had grade 3 tumors; and one case had an unknown grade.

Diagnostic biopsies only were performed in 15 of 27 patients (55.6%; missing information in one patient). The primary pancreatic lesion served as the biopsy site in eight patients, while tissue sampling from distant metastatic sites was conducted in six patients. Biopsy site documentation was unavailable for one patient. In all 15 patients, panNET diagnosis was made on the biopsy specimen.

### 3.3 Treatment of 28 children and adolescents with pancreatic NET

Surgical resection was performed in 18 of 28 patients (64.3%) including pancreas tail resection (*N* = 10), classic Whipple procedure (*N* = 3), pylorus‐preserving pancreas head resection (*N* = 2), and pylorus‐preserving duodenopancreatectomy (*n* = 2). (Figure 1) Details on the surgical procedure performed were missing in one patient. The selection of surgical procedures was guided by tumor localization and disease extent. Among surgically treated patients, complete resection with negative margins (R_0_) was achieved in 13 patients (72.2%); microscopically (R_1_) or macroscopically (R_2_) positive resection margins were achieved in two patients each (Table 2). One patient had an indeterminate (R_x_) resection margin. Subsequent surgical interventions were performed in three patients (16.6%).

**TABLE 2:** Tumor characteristics in 28 patients with pancreatic neuroendocrine tumors.

Characteristics	German MET registry	
	Count	Proportion, %
Tumor size, histologically	18	100
Median, cm	5.5 (range, 0.5–17.0)	
Size ≤5 cm	8	44.4
Size >5 cm	9	50
Unknown	1	5.6
pT stage		
T1	1	5.6
T2	5	27.8
T3	9	50
T4	2	11.1
Tx	1	5.6
c/pN stage		
N0	14	53.8
N1	12	42.3
Unknown	2	7.1
cM stage		
M0	12	44.4
M1	15	55.6
Unknown	1	3.6
R classification		
R0	13	72.2
R1/R2	4	22.2
Rx	1	5.6
WHO classification of panNET^a^, ^b^		
Well‐differentiated NET		
G1 (Ki67 <3%, mitotic index <2)	4	14.3
G2 (Ki67 3%–20%, mitotic index 2–20)	12	42.9
G3 (Ki67 >20%, mitotic index >20)	2	11.1
Poorly differentiated NEC		
High (Ki67 >20%, mitotic index >20)	7	25.0
Unknown	3	10.7
Ki67 index		
≤10%	13	46.4
>10%	11	39.3
Unknown	4	14.3

^a^ According to the WHO classifications of 2017 [14], 2019 [15], and 2022 [16], in case the Ki67 index and mitotic index showed discrepant results, the index with the highest grade was used for classification.

^b^ Including histopathological results from biopsies only.

Histopathological assessment of the surgical specimens or biopsy materials demonstrated well‐differentiated, grade 1–3 NETs in 18 of 25 patients (72.0%; missing information in three patients). Poorly differentiated neuroendocrine carcinomas were identified in seven patients (28.0%) (Table 2).

Among the 10 patients presenting with panNETs confined to the pancreas, surgical resection was performed in nine cases (90%; missing information in one patient). Two of these patients underwent subsequent surgical procedures for complications. No adjuvant therapy was administered in this subgroup. Histopathological evaluation demonstrated regional lymph node involvement in one patient.

Both patients (100%) clinically diagnosed with (isolated) lymph node involvement underwent surgical resection. Regional lymph node involvement was confirmed by histopathological evaluation in only one of these patients.

Of the 15 patients with distant metastases, nine (60.0%) received neoadjuvant chemotherapy aimed at improving surgical resectability. Treatment regimens are detailed in Table 3. Response assessment demonstrated complete remission in one patient, stable disease in two patients, and disease progression in one, with two patients lacking documented response evaluation. Following neoadjuvant therapy, three patients proceeded to surgical resection.

**TABLE 3:** Details on systemic therapies in children and adolescents with pancreatic neuroendocrine tumors.

Age at Diagnosis (years), sex	Ki67 (%)	Metastases	Neoadjuvant chemotherapy (cycles)	Surgical re‐section	First‐line/adjuvant therapy (cycles)	Outcome/follow‐up (years)
15.0, m	50	Liver, lymph nodes	MI/MII (4x)	R0	MI/MII (4x)	Alive in 1. CR FU: 3.8
14.7, m	90	Liver	(1) Oxaliplatin/irinotecan/gemcitabine (7x) (2) Ifosfamide/vincristine/actinomycin D/doxorubicin (3x)	–	–	Alive with progressive disease FU: 1.3
14.7, m	20	Lung	(1) Etoposide/carboplatin (4x) (2) Carboplatin (2x) + radiotherapy (3) Etoposid/carboplatin (1x) (4) Pegylated interferon (7x)	–	–	Deceased after local tumor relapse Deceased: 2.6
16.8, m	5	Distant lymph nodes	–	R2	(1) MI/MII (3x) (2) MIBG‐therapy (3) Sandostatin (4) Capecitabine (5) DOTATOC (1x) (6) Capecitabine/ bevacizumab	Death from tumor progression Deceased: 2.8
13.8, f	10	Liver, lymph nodes	MI/MII (5x) Sandostatin	–	–	Death from tumor progression Deceased: 1.7
12.9, f	20	Liver, lymph nodes	(1) Oxaliplatin/irinotecan/gemcitabine (6x) (2) Cisplatin/etoposide/ifosfamide (2x)	–	(1) Oxaliplatin/irinotecan/gemcitabine (6x) (2) Cisplatin/etoposide/ ifosfamide (2x) (3) DOTATOC (7x)	Death from tumor progression Deceased: 10.3
14.3, f	15	Liver, lung, ovaries, bones, lymph nodes	Cisplatin/etoposide (1x)	R2	(1) DOTATOC (4x) (2) 5‐FU/temozolamide (3) Everolimus (4) Radiotherapy	Death from tumor progression Deceased: 2.2
14.3, f	25	Liver, lymph nodes	(1) Temozolamide/thalidomide (2) DOTATOC (4x)	R2	(1) Sunitinib (2) Lanreotide (3) DOTATOC(1x) (4) Radiotherapy	Alive with progressive disease after distant relapse FU: 5.9
15.2, f	7	Liver	–	Rx	Everolimus	Alive in stable disease after relapse of liver metastases LFU: 4.3
17.9, f	G3/25	Liver, bones, lymph nodes	–	R0	(1) Cisplatin/etoposide (2x) (2) Capecitabine/temozolomide (2x) (3) DOTATOC (4x) + Capezitabine/temzolomid (4) Streptozotocin/5‐FU (3x)	Deceased from progression of liver metastases Deceased: 5.6
15.2, f	G2/15	Liver, lymph nodes	Not detailed	–	(1) Everolimus (2) Capecitabine/temozolomid	Alive in stable disease FU: 1.5
17.1, m	G3/90	Liver, lymph nodes	(1) Irinotecan/cisplatin (6x) (2) Capecitabine/temozolamide (2x)	–	–	Death from tumor progression Deceased: 0.7

*Note*: MI: vincristine 1.5 mg/m^2^ day 1 + 8, etoposide 100 mg/m^2^ day 1–4, cisplatin 40 mg/m^2^ day 1–4; and MII: vindesine 3 mg/m^2^ day 1 + 8, dacarbazine 200 mg/m^2^ day 1–4, ifosfamide 1500 mg/m^2^ day 1–4, doxorubicin 35 mg/m^2^ day 4 + 5.

Abbreviations: CR, complete remission; hist., histological; LFU, last follow‐up; n.a., not applicable; neg, negative; PD, progressive disease; pos, positive; PR, partial remission; SD, stable disease; SRI, somatostatin receptor imaging.

Of the 15 patients with distant metastatic disease, seven (46.7%) underwent surgical resection. R_0_ resection of the primary was achieved in two patients, though the hepatic metastases in one case remained surgically unaddressed. Microscopically or macroscopically positive resection margins were documented in three patients, including two who had received neoadjuvant chemotherapy. One patient had indeterminate resection margin status. Hepatic metastasectomy was performed in four patients, with one case additionally involving resection of the ovarian metastases.

First‐line or adjuvant treatment was administered to 8 of 15 patients (53.3%). The therapeutic strategy consisted of therapy with MI/MII cycles in two patients, while seven patients received combination therapy. The multimodal treatment approaches included other chemotherapies (*n* = 5), [^177^Lu]Lu DOTATATE therapy (*n* = 5), and radiotherapy (*n* = 2). Additional therapeutic interventions comprised mTOR inhibition with everolimus in three patients and somatostatin analog administration for symptom control in two patients. Table 3 provides comprehensive details of the systemic therapeutic regimens employed.

### 3.4 Survival outcomes and risk factors associated with poor outcomes

**FIGURE 2:**
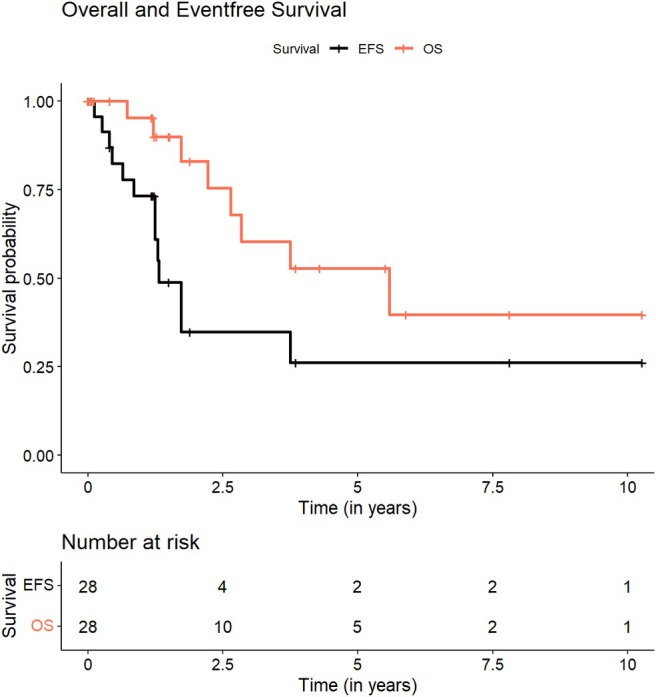
Overall and event‐free survival in 28 pediatric patients with pancreatic NET.

**FIGURE 3:**
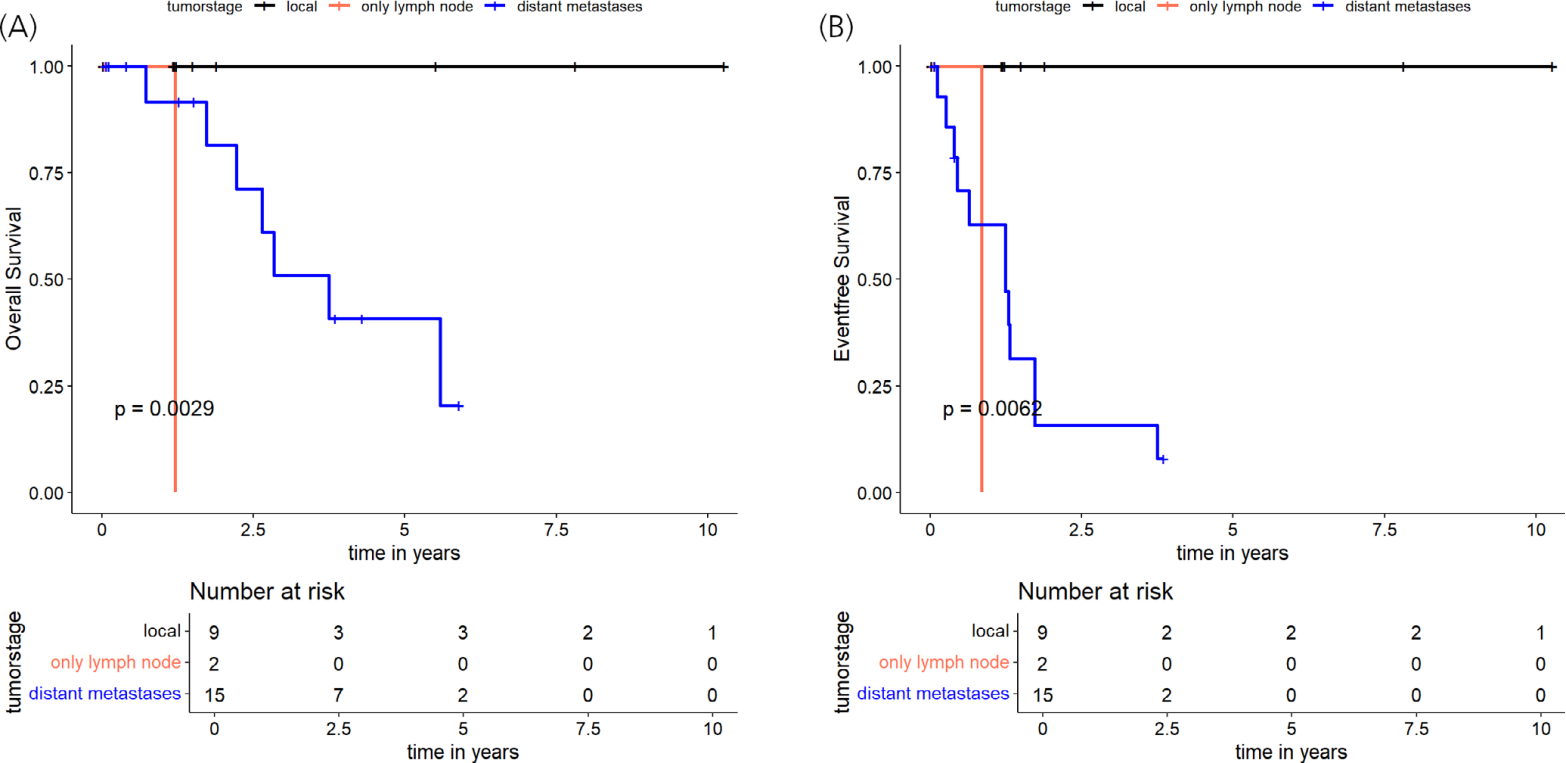
Overall (A) and event‐free survival (B) in 28 pediatric patients with localized disease, isolated lymph node involvement, and distant metastases based on clinical and histopathological assessment, respectively.

At a median follow‐up duration of 1.5 years (range, 0–10.3 years), the Kaplan–Meier estimates at 1‐year and 3‐year OS rates were 95.2% (95% CI: 86.6%–100%) and 60.4% (95% CI: 39.6%–92%), respectively (Figure 2). Analysis stratified by disease stage revealed significantly different outcomes, with 3‐year OS rates of 100% for localized disease compared to 50.9% for patients with distant metastases (*p* = .0029) (Figure 3A).

EFS analysis revealed 1‐year and 3‐year rates of 73.2% (95% CI: 57%–94.1%) and 34.9% (95% CI: 17.9%–67.8%), respectively (Figure 2). The median time to event was 15.7 months. Disease stage significantly influenced EFS, with 3‐year rates of 100% for localized compared to 15.7% for distant metastatic disease (*p* = .0062) (Figure 3B).

Radiological assessment at the last follow‐up demonstrated complete remission in 12 of 28 patients (42.9%; localized disease *n* = 9, isolated lymph node involvement *n* = 1, distant metastases *n* = 2), while stable disease was maintained in four patients (14.3%) for periods ranging from 0.1 to 4.3 years. Disease progression was documented in two patients (7.7%) with distant metastases. Fatal outcomes were observed in eight patients (28.6%) with a median interval from diagnosis to death of 2.4 years (range, 0.7–5.6 years). Treatment response data were unavailable for two patients.

Complete remission remained unachieved in 11 of 26 patients (42.3%; missing information in two patients), comprising ten patients with distant metastases and one patient with isolated lymph node involvement. Recurrences were observed in three patients (11.5%), presenting as local recurrence, lymph node relapse, and distant relapse, respectively. These patients had initially presented with pT3N1M0, pT3N1M1, and pT2N0M1 disease. The recurrences occurred 0.8, 1.7, and 0.6 years after complete remission, respectively. One patient with von Hippel–Lindau syndrome developed a subsequent pheochromocytoma 1.2 years following the initial panNET diagnosis.

Statistical analysis revealed significant associations between EFS and metastatic disease (M0 vs. M1, *p* = .0082), as well as surgical resection status (R_0_ vs. R_1/2_ vs. no resection, *p* = .0077). No significant correlations were observed for lymph node status (N0 vs. N1, *p* = .12), sex (female vs. male, *p* = .22), tumor localization within the pancreas (head vs. body vs. tail, *p* = .86), extent of the primary tumor (pT1‐2 vs. pT3‐4, *p* = 1.0), tumor size (≤5 cm vs. >5 cm, *p* = .76), pathological grade (G1 vs. G2‐3, *p* = .28), or proliferation index (Ki67 ≤10% vs. >10%, *p* = .11).

## 4 DISCUSSION

Our analysis of 28 pediatric patients with panNETs represents one of the most comprehensive datasets for this rare malignancy in children and adolescents. The findings provide valuable insights into clinical presentation, treatment strategies, and outcomes while emphasizing the differences between pediatric and adult cases and underscoring the need for tailored guidelines.

The classification of panNETs has evolved significantly over time, introducing challenges in defining and categorizing these tumors, particularly with regard to malignancy.^2^ Early frameworks, such as Williams and Sandler's embryological classification (1963), highlighted the distinct clinical behavior of foregut, midgut, and hindgut neuroendocrine tumors.^17^ The WHO classification (2000–2004) subsequently introduced biological behavior‐based categories, distinguishing well‐differentiated endocrine tumors (benign or uncertain behavior) from poorly differentiated carcinomas with high‐grade malignancy.^18^, ^19^, ^20^ More recent advances, such as the ENETS guidelines, have added grading systems based on the Ki67 index and mitotic count, further refining prognostic stratification.^21^, ^22^ However, these systems, while validated in adults, remain underexplored in pediatric populations, complicating accurate classification and risk assessment.

In our cohort, the predominance of nonfunctional panNETs (75%) diverges from the literature, where insulinomas often dominate pediatric cases, representing up to 70%–85%.^8^ This discrepancy may reflect a selection bias within the German MET Registry, which primarily includes malignant cases, as insulinomas are typically considered benign.^2^ Additionally, limited use of preoperative biochemical diagnostics may have contributed to underreporting functional tumors, highlighting the diagnostic complexities in pediatric panNETs. A systematic approach to biochemical and imaging evaluations is essential to improve classification accuracy and clinical outcomes.

Our findings strongly support the critical role of surgical resection in improving outcomes for pediatric panNETs. Patients with localized disease achieved a 3‐year OS of 100%, consistent with SEER data reporting 5‐year OS rates exceeding 80% for localized cases.^3^, ^23^, ^24^ Conversely, metastatic cases exhibited a 3‐year OS of 50.9%, aligning with previous studies reporting median OS estimates of approximately 40%.^25^ Achieving R0 resection was a key prognostic factor, reinforcing its pivotal role in survival.^6^, ^7^, ^23^ These results underscore the need for early detection and aggressive surgical management to optimize outcomes, particularly given the absence of robust, pediatric‐specific treatment guidelines.^21^, ^22^

Pediatric panNETs exhibit distinct biological and clinical features compared to adult cases, necessitating tailored management approaches. A higher prevalence of hereditary syndromes, such as MEN1, was observed in our cohort (17.9%), consistent with prior reports suggesting that up to 25% of pediatric panNETs are hereditary.^12^, ^26^ This underscores the importance of systematic genetic screening to inform surveillance and management strategies. Molecular profiling, including *ATRX*/*DAXX* mutations, alternative lengthening of telomeres (ALT), and somatostatin receptor (SST2) expression, remains well‐characterized in adults but is underexplored in pediatric cases.^27^ Incorporating these markers into pediatric research could refine prognostic models and identify novel therapeutic targets.

The ENETS guidelines for adult panNETs emphasize site‐specific TNM staging, a three‐tiered grading system, and tailored treatment strategies based on functionality and disease stage. However, their direct applicability to pediatric cases remains uncertain, further highlighting the need for pediatric‐specific guidelines developed through multicenter collaborations.^21^, ^22^

Several limitations warrant consideration. First, the small sample size reflects the rarity of pediatric panNETs but limits the generalizability of our findings and multivariate analysis. Second, incomplete genetic and molecular profiling precludes a comprehensive understanding of tumor biology. Third, data incompleteness, particularly for benign or asymptomatic cases, may introduce systemic bias. Finally, treatment heterogeneity across centers underscores the need for international trials and standardized protocols to harmonize management strategies and improve outcomes.

Our findings highlight several actionable areas for advancing the management of pediatric panNETs.

1. Establishing pediatric‐specific guidelines informed by multicenter studies is critical to standardize treatment.
2. Routine genetic screening should be integrated into clinical workflows to identify hereditary syndromes and inform surveillance strategies.
3. Expanding molecular profiling studies to investigate biomarkers such as *ATRX*/*DAXX* mutations and somatostatin receptor expression could unlock novel therapeutic targets and refine prognostic models.
4. Structured long‐term follow‐up programs are essential to monitor late effects, recurrence, and overall quality of life in these patients.

## 5 CONCLUSION

Disease stage and surgical resection are critical prognostic factors in pediatric panNETs. Localized disease demonstrated excellent survival rates, while metastatic cases underscore the need for improved systemic therapies. Pediatric‐specific management guidelines are necessary, incorporating genetic screening and molecular profiling into routine practice.

## AUTHOR CONTRIBUTIONS

**Katharina Karges:** Data curation; formal analysis; writing – original draft; visualization. **Marina Kunstreich:** Investigation; data curation; project administration; writing – review and editing. **Ulrich‐Frank Pape:** Writing – review and editing. **Jörg Fuchs:** Data curation; investigation; writing – review and editing. **Christian Vokuhl:** Investigation; writing – review and editing. **Michael Abele:** Writing – review and editing; investigation; project administration. **Dominik T. Schneider:** Investigation; writing – review and editing; project administration. **Ines B. Brecht:** Investigation; writing – review and editing; project administration. **Constantin Lapa:** Writing – review and editing; data curation. **Michael C. Frühwald:** Writing – review and editing; investigation. **Peter Vorwerk:** Investigation; writing – review and editing; funding acquisition; conceptualization; project administration; resources. **Antje Redlich:** Conceptualization; investigation; funding acquisition; writing – review and editing; project administration; resources; data curation. **Michaela Kuhlen:** Conceptualization; investigation; funding acquisition; writing – original draft; methodology; formal analysis; data curation; resources; supervision; project administration; writing – review and editing.

## FUNDING INFORMATION

The German MET studies were funded by Deutsche Kinderkrebsstiftung, grant number DKS 2014.06, DKS 2017.16, DKS 2021.11, DKS 2024.16, Mitteldeutsche Kinderkrebsforschung, and Magdeburger Förderkreis krebskranker Kinder e.V.

## CONFLICT OF INTEREST STATEMENT

The authors have no conflicts of interest to declare.

## PEER REVIEW

The peer review history for this article is available at https://www.webofscience.com/api/gateway/wos/peer-review/10.1111/jne.70039.

## ETHICS STATEMENT

The GPOH‐MET 97 protocol, the GPOH‐MET 2013 registry, and the MET registry were approved by the ethics committees of the University of Luebeck (Approval number 97125) and the Otto‐von‐Guericke‐University Magdeburg (Approval numbers 174/12 and 52/22), Germany.

## PATIENT CONSENT STATEMENT

Written informed consent was obtained from patients and/or legal guardians as appropriate.

## Supporting information


**Table S1.** Diagnostic details for pancreatic neuroendocrine tumors.

## Data Availability

The data that support the findings of this study are available on request from the corresponding author. The data are not publicly available due to privacy or ethical restrictions.
