# Supplementary material for: Pancreatic neuroendocrine tumors in children and adolescents—Data from the German MET studies (1997–2023)
Source: J Neuroendocrinol. 2025 May 11;37(8):e70039. doi: 10.1111/jne.70039 (PMC12358202; doi:10.1111/jne.70039)
Supplement: Supplementary file 1 — Table S1. Diagnostic details for pancreatic neuroendocrine tumors. [file JNE-37-e70039-s001.docx]

**Supplemental Table 1.** Diagnostic details for pancreatic neuroendocrine tumors.

| **Sub-entity** | **Clinical Presentation** | **Biochemical Markers** | **Imaging Techniques** | **Histopathological Features** |
| --- | --- | --- | --- | --- |
| Insulinoma | Hypoglycemia, seizures | Elevated insulin, C-peptide | CT, MRI, endoscopic ultrasound | Well-differentiated, insulin-positive cells |
| Gastrinoma | Peptic ulcers, diarrhea | Elevated gastrin | CT, MRI, somatostatin receptor scintigraphy (SRS) | Gastrin-positive cells |
| Glucagonoma | Diabetes, dermatitis | Elevated glucagon | CT, MRI, PET | Glucagon-positive cells |
| VIPoma | Watery diarrhea | Elevated VIP | CT, MRI, PET | VIP-positive cells |
| Non-functioning panNET | Abdominal mass, pain | Normal hormone levels | CT, MRI, PET | Lacks hormone production, neuroendocrine markers |
